# Targeting CD19 in diffuse large B‐cell lymphoma: An expert opinion paper

**DOI:** 10.1002/hon.3013

**Published:** 2022-06-07

**Authors:** Sarah Bailly, Guillaume Cartron, Sridhar Chaganti, Raul Córdoba, Paolo Corradini, Johannes Düll, Isacco Ferrarini, Wendy Osborne, Andreas Rosenwald, Juan‐Manuel Sancho, Hervé Tilly, Eric Van Den Neste, Andreas Viardot, Carlo Visco

**Affiliations:** ^1^ Département d’Hématologie Cliniques Universitaires Saint‐Luc Brussels Belgium; ^2^ Department of Haematology Centre Hospitalier Universitaire de Montpellier UMR‐CNRS 5535 Montpellier France; ^3^ Department of Haematology Queen Elizabeth Hospital Birmingham UK; ^4^ Department of Hematology Fundación Jiménez Díaz University Hospital Health Research Institute IIS‐FJD Madrid Spain; ^5^ Fondazione IRCCS Istituto Nazionale dei Tumori University of Milan Milan Italy; ^6^ Medizinische Klinik und Poliklinik II Universitätsklinikum Würzburg Würzburg Germany; ^7^ Department of Medicine Section of Hematology University of Verona Verona Italy; ^8^ Newcastle Upon Tyne Hospitals NHS Foundation Trust Newcastle UK; ^9^ Institute of Pathology University of Würzburg, and Comprehensive Cancer Center Mainfranken Würzburg Germany; ^10^ ICO‐IJC‐Hospital Germans Trias I Pujol Barcelona Spain; ^11^ Department of Hematology and U1245 Centre Henri Becquerel and University of Rouen Rouen France; ^12^ Department of Internal Medicine III University Hospital Ulm Ulm Germany

**Keywords:** CD19, diffuse large B‐cell lymphoma, non‐Hodgkin lymphoma, relapsed/refractory

## Abstract

The ubiquitous, early‐stage expression, efficient internalization, limited off‐target effects, and high disease specificity of CD19 make it an attractive therapeutic target. Currently available anti‐CD19 therapies have demonstrated particular promise in patients with relapsed or refractory B‐cell non‐Hodgkin lymphoma. Selection of the most appropriate treatment strategy should be based on individual patient characteristics and the goal of therapy. However, evidence and knowledge about the sequencing of anti‐CD19 therapies are limited. Here, we review the current evidence for CD19 as a target in diffuse large B‐cell lymphoma and consider approaches to the use of anti‐CD19 therapy.

## 1 INTRODUCTION

Diffuse large B‐cell lymphoma (DLBCL) is the most common type of non‐Hodgkin lymphoma (NHL), constituting around 25%–40% of cases worldwide.^1^, ^2^ The annual incidence of DLBCL is three to four per 100,000 persons in Europe and 7 per 100,000 persons in the United States,^3^, ^4^ with diagnosed incident cases predicted to increase steadily over the coming years.^5^

The introduction of the anti‐CD20 monoclonal antibody rituximab in 1997 revolutionized the treatment of DLBCL.^6^ Rituximab‐mediated cell death is thought to occur through several different mechanisms including natural killer (NK)‐cell–mediated antibody‐dependent cellular cytotoxicity (ADCC), antibody‐dependent cellular phagocytosis (ADCP), complement‐dependent cytotoxicity (CDC), and direct antitumor effects via apoptosis or other cell death pathways.^7^, ^8^ Rituximab given in combination with cyclophosphamide, doxorubicin, vincristine, and prednisone (R‐CHOP) was approved in 2006 as first‐line treatment for patients with DLBCL and remains the standard therapy for previously untreated patients, achieving complete and sustained remission in approximately 60% of this population.^9^, ^10^, ^11^ However, around 30%–40% of patients relapse following an initial response to therapy or are unable to achieve remission with first‐line treatment.^1^ Among patients with relapsed/refractory (R/R) DLBCL, 30%–40% have a response to salvage chemotherapy and up to 50% of these patients subsequently undergo autologous stem‐cell transplantation (ASCT), with around half ultimately relapsing after transplantation.^1^, ^3^

Patients with R/R DLBCL currently have few treatment options, and outcomes are poor due to a lack of durable response to salvage chemotherapy.^12^ Rituximab‐containing regimens are frequently used, despite limited evidence supporting their use.^2^ Various mechanisms of resistance to rituximab have been proposed,^13^ with CD20‐negative change after rituximab‐containing therapy believed to be one of the main resistance mechanisms in B‐cell NHLs. This is thought to occur as a result of epigenetic downregulation of CD20 messenger ribonucleic acid expression, homozygous deletion of the CD20 gene, and selection of CD20‐negative clones.^14^ Research suggests that many patients with DLBCL may have a low peripheral blood NK cell count, and this may be associated with poorer outcomes with anti‐CD20–based therapy, including shorter progression‐free survival (PFS), versus patients who have higher NK cell counts.^15^

A considerable focus has been placed on improving outcomes of first‐line treatment and increasing cure rates in newly diagnosed DLBCL,^16^ although there is limited evidence demonstrating a consistent, clear benefit compared with R‐CHOP. Preliminary results from the POLARIX trial suggest that treatment with polatuzumab vedotin in combination with R‐CHOP improves PFS compared with standard of care R‐CHOP in patients with DLBCL, but further data are awaited to confirm this.^17^ Efforts to improve outcomes for patients with R/R DLBCL are also ongoing through the development of novel agents with new targets, including the B‐cell surface antigens CD19, CD22, CD37, and CD79B.^18^ Among these, CD19 shows particular promise, since it is constantly and strongly expressed in the vast majority of B‐cell lymphoproliferative diseases, is highly disease‐specific, and has a limited number of off‐target effects.^19^, ^20^

In this paper, we examine the role of CD19 in the pathophysiology of DLBCL, review the evidence for CD19 as a target in DLBCL, and consider approaches to the use of anti‐CD19 therapy.

## 2 NATURAL ROLE OF CD19 AND ROLE IN B‐LYMPHOMAGENESIS

**FIGURE 1:**
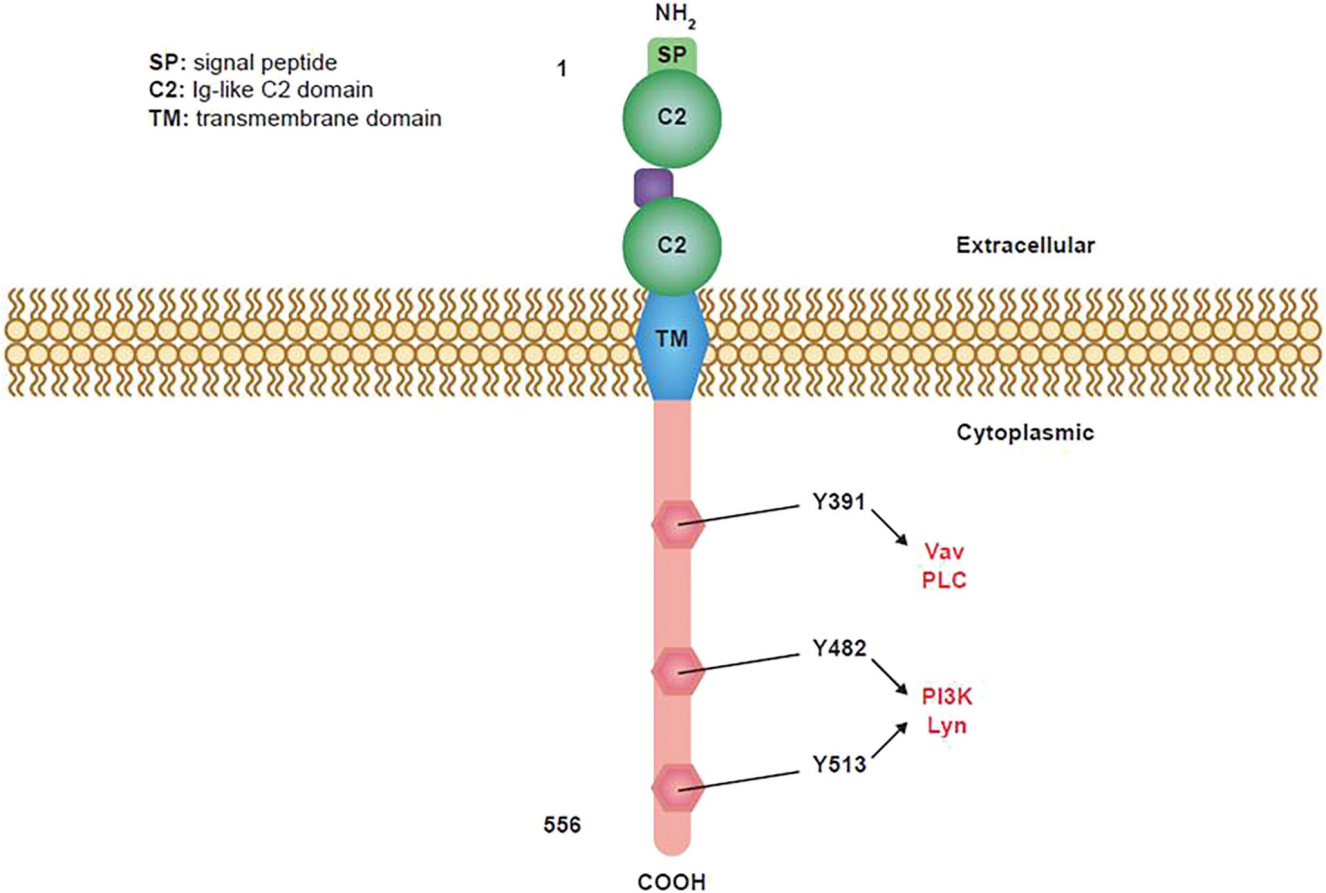
Molecular structure of CD19^22^ CD19 is a type I one‐pass transmembrane protein. The two extracellular C2 Ig‐like domains are separated by a small helical non‐Ig domain with possible disulfide links. The highly conserved, 242 amino acid cytoplasmic domain includes multiple tyrosine residues. Three key tyrosine residues are shown with their associated signaling kinases and molecules. Ig, immunoglobulin; PI3K, phosphoinositide 3‐kinase; PLC, phospholipase C. Figure reproduced with permission from BioMedCentral from Wang K, Wei G, Liu D. CD19: a biomarker for B cell development, lymphoma diagnosis and therapy. *Exp Hematol Oncol*. 2012;1(1):36.

CD19 is a type 1 transmembrane glycoprotein that belongs to the immunoglobulin (Ig) superfamily.^21^, ^22^ It consists of a single transmembrane domain, a cytoplasmic C‐terminus, and extracellular N‐terminus (Figure 1).^22^ CD19 is the main signaling component of a multimolecular complex located on the surface of mature B cells together with tetraspanin membrane protein CD81, complement receptor CD21, and CD225.^22^, ^23^, ^24^, ^25^ CD81 is required for the transfer of CD19 to the cell surface and for CD19 surface expression.^26^, ^27^, ^28^, ^29^, ^30^ CD19 is expressed ubiquitously on the surface of B cells from the early to mature stages of development,^31^ and is subsequently downmodulated at the plasma cell stage.^23^, ^32^ CD19 is not shed from the surface of B cells into the circulation.^33^ The expression profile of CD19 is broader than that of CD20^34^ and CD19 is expressed at an earlier pre‐B stage than CD20.^35^ In normal cells, CD19 plays a key role in many B‐cell functions, including development and differentiation, proliferation, and signaling.^23^, ^36^

CD19 also modulates B‐cell receptor signaling at several stages of B‐cell development, and effects antigen‐independent and immunoglobulin‐induced B‐cell activation through various protein kinases.^22^, ^37^

B‐cell receptors consist of a surface‐expressed immunoglobulin joined to CD79A and CD79B heterodimers, which are necessary for signal transduction, plasma membrane expression, and intracellular trafficking.^38^, ^39^ In DLBCL, B‐cell receptor expression is retained, and these receptors play a pivotal role in lymphoma pathogenesis and proliferation.^38^, ^39^ The activated B‐cell–like DLBCL subtype is characterized by chronic active B‐cell receptor signaling and activation of the Nuclear Factor Kappa B signaling pathway, and mainly expresses immunoglobulin M (IgM)‐B‐cell receptors.^38^, ^39^ The germinal center B‐cell–like subtype expresses IgG‐B‐cell receptors.^39^ IgM‐B‐cell receptor signaling preferentially induces pro‐survival and mitogenic signals, while IgG‐B‐cell signaling favors plasma cell differentiation.^39^

The B‐cell–specific paired box transcription factor 5 (PAX5) plays a role in B‐cell development and normal expression of CD19.^35^ PAX5 and CD19 regulate levels of the multifunctional transcription factor, c‐MYC, through a post‐transcriptional mechanism that acts via the phosphoinositide 3‐kinase pathway, independently of B‐cell receptor activity.^35^ Increased expression of c‐MYC leads to uncontrolled cell growth and tumor development. A c‐MYC–CD19 regulatory loop, in which CD19 and c‐MYC act synergistically, accelerates B‐cell lymphomagenesis and lymphoma progression.^31^, ^35^

## 3 EVIDENCE OF CD19 AS A PROMISING TARGET IN DIFFUSE LARGE B‐CELL LYMPHOMA

Initial attempts to target CD19 through the development of conventional anti‐CD19 monoclonal antibodies were unsuccessful, due to a lack of efficacy of these first‐generation therapies that retain the native Fc region of immunoglobulin G1 (IgG1).^34^, ^40^ Native CD19 antibodies containing unmodified Fc regions cannot elicit key antibody effector functions, because they do not induce growth arrest or programmed cell death, and have limited effectiveness in triggering CDC, ADCC and ADCP.^40^ This prompted the development of novel anti‐CD19 targeted‐immunotherapy strategies, which have demonstrated particular promise in B‐cell NHL.

### 3.1 Fc‐modified anti‐CD19 monoclonal antibodies: tafasitamab

Evidence demonstrating the significance of antibody functions dependent on the Fc domain provided a rationale for Fc engineering, to generate anti‐CD19 antibodies with enhanced efficacy.^40^

Tafasitamab is an Fc‐modified IgG1, humanized, anti‐CD19 monoclonal antibody that binds to CD19 expressed on the surface of pre‐B and mature‐B lymphocytes. It acts by mediating ADCC and ADCP, and exerts a direct cytotoxic effect.^41^, ^42^ Two amino acid substitutions have been engineered in the Fc region at S239D and I332E, meaning that tafasitamab has an increased affinity for Fc gamma receptors, including FcγRIIIa (expressed on the surface of NK cells, macrophages, and many γδ T cells), and enhanced FcγRIIIa‐mediated ADCC and ADCP activity.^41^, ^43^

Tafasitamab has shown potent activity in preclinical studies involving lymphoma and leukemia models, including 100‒1000‐fold enhanced ADCC relative to an anti‐CD19 IgG1 analog in vitro.^41^, ^42^ Tafasitamab has demonstrated single‐agent activity in the setting of R/R DLBCL and other B‐cell malignancies, with some patients achieving durable responses.^44^, ^45^, ^46^, ^47^ Preclinical evidence also suggests that tafasitamab acts synergistically in combination with lenalidomide.^42^ Lenalidomide has direct antineoplastic activity and indirect effects mediated via various immune cells in the tumor microenvironment.^48^ In particular, lenalidomide stimulates NK‐cell proliferation and activation, thus enhancing NK‐cell–mediated ADCC by tafasitamab in vitro.^42^, ^48^

Phase one studies of tafasitamab have provided data on its safety, efficacy, pharmacokinetics and appropriate dosing to support phase two development.^49^, ^50^ First‐MIND (NCT04134936), an ongoing phase 1b open‐label trial investigating the safety and efficacy of tafasitamab plus R‐CHOP or tafasitamab and lenalidomide plus R‐CHOP in adult patients with newly diagnosed DLBCL, found no unexpected toxicity and no effect on the relative dosage intensity of R‐CHOP with the addition of both therapies. Preliminary efficacy analyses showed a combined overall response rate (ORR) of 83.4% and complete remission rate of 75.0%.^49^

The phase two L‐MIND trial (NCT02399085) investigated the combination of tafasitamab plus lenalidomide followed by tafasitamab monotherapy in 80 patients with DLBCL who relapsed or were refractory after one to three systemic regimens (including at least one CD20‐targeting regimen), and who were ineligible for ASCT. Exclusion criteria included primary refractory DLBCL, defined as no response to, or progression during or within 6 months of, frontline therapy. Fifteen patients (19%) with primary refractory disease were included in the study. Prior to a protocol amendment, patients who relapsed within 3 months of a prior anti‐CD20‐containing regimen were defined as primary refractory and were excluded from L‐MIND. Patients who relapsed or progressed between 3 and 6 months after receiving frontline therapy recruited before the amendment were considered as primary refractory but were included. In total, 42.0% and 44.4% of patients were refractory to rituximab and last line of therapy, respectively. The median number of prior lines of systemic therapy was two, with 50% of patients (*n* = 40) receiving tafasitamab as second‐line therapy. A history of double‐/triple‐hit lymphoma was an exclusion criterion of the study; however, two patients were found to have these alterations post‐enrollment.^45^

Any grade treatment‐emergent adverse events (TEAEs) occurred in 100% of patients. The most common grade 3 or higher TEAEs with the combination of tafasitamab and lenalidomide were neutropenia (48%), thrombocytopenia (17%), and febrile neutropenia (12%). Infusion‐related reactions (all grade 1 and during the first infusion) were reported in 6% of patients, none of which required treatment discontinuation. Serious adverse events were reported in 51% of patients and 25% of patients discontinued treatment due to adverse events.^45^

At ≥35 months of follow up, the ORR was 57.5% (46 patients) and 40.0% (32 patients) achieved a complete response (CR). The median duration of response (DOR) was 43.9 months and median PFS was 11.6 months with a median follow‐up of 33.9 months. Median overall survival (OS) was 33.5 months with a median follow‐up of 42.7 months.^47^ Median PFS was 23.5 months in patients who received tafasitamab plus lenalidomide as second‐line therapy compared with 7.6 months in those who received the combination as third‐line or later. Median OS was 45.7 versus 15.5 months, respectively.^51^

In a *post‐hoc* subgroup analysis of patients in L‐MIND who were refractory to primary therapy (*n* = 15) or their last line of therapy (*n* = 35), ORRs were similar to non‐refractory patients (both 60.0%). The 12‐month DOR was similar regardless of refractory status to last therapy, although the 12‐month PFS and OS were lower compared with non‐refractory patients.^52^ Responses were observed in two patients who had double‐ or triple‐hit lymphoma.^45^ In a patient‐level matched comparison of the L‐MIND and real‐world RE‐MIND cohorts of patients with ASCT‐ineligible R/R DLBCL (76 patients each cohort), the combination of tafasitamab and lenalidomide resulted in a significantly better ORR, CR, and OS compared with lenalidomide monotherapy (ORR 67.1% vs. 34.2%; odds ratio 3.89; 95% confidence interval 1.90–8.14; *p < *0.0001).^53^

Tafasitamab received accelerated approval from the US Food and Drug Administration (FDA) in July 2020 for use in combination with lenalidomide to treat transplant‐ineligible adults with R/R DLBCL.^54^, ^55^ The European Medicines Agency granted marketing authorization for tafasitamab on 26 August 2021.^56^ Studies are ongoing to evaluate the use of tafasitamab as first‐line treatment for DLBCL (First‐MIND [NCT04134936^57^] and Front‐MIND [NCT04824092^58^]), and in R/R follicular lymphoma or marginal zone lymphoma (InMIND [NCT04680052^59^]).

### 3.2 Anti‐CD19 bispecific T‐cell engagers: blinatumomab

Strategies to target CD19 focusing on T cell recruitment led to the development of blinatumomab, a CD19xCD3 bispecific T‐cell engager that directs cytotoxic T‐cells to lyze CD19‐expressing B cells.^60^, ^61^, ^62^, ^63^ Blinatumomab exhibits linear pharmacokinetics and due to its short elimination half‐life (*t*_1/2_ 1–2 h), must be administered as a 24‐h continuous intravenous infusion.^64^ Blinatumomab has demonstrated activity in three phase 1/2 trials in heavily pre‐treated patients with aggressive R/R DLBCL, with CR rates ranging from 19% to 22%.^62^, ^65^ In a pooled analysis of these studies, the median duration of CR and OS was not reached in patients who achieved CR or complete metabolic response within cycle 1 (12 weeks) of blinatumomab treatment (*N* = 17), with a median follow‐up of 15.6 and 16.4 months, respectively. At 12 months, 62.2% of patients were still responding and 86.5% were alive.^60^ In a single‐center, long‐term follow‐up study of blinatumomab in patients with R/R B‐NHL from the phase one MT103‐104 trial, the median OS was 5.8 years in patients who received a dose of ≥60 μg/m^2^ per day (*n* = 25). Median PFS was 1.5 years and treatment‐free survival was 3.5 years.^66^

Neurologic adverse events, most commonly dizziness, tremor, confusional state, and encephalopathy are reported in around half of patients treated with blinatumomab and frequently lead to dose interruption and treatment discontinuation.^67^

### 3.3 Anti‐CD19 antibody–drug conjugates: loncastuximab tesirine

The characteristics of CD19 (i.e., rapid internalization kinetics, broader expression profile than CD20, not shed into the circulation) make it a better target for antibody–drug conjugates (ADCs) than CD20.^34^, ^68^ Loncastuximab tesirine is an ADC consisting of a CD19‐targeting antibody conjugated to a cytotoxic DNA minor groove interstrand cross‐linking pyrrolobenzodiazepine dimer.^69^, ^70^ After binding to CD19‐positive cells, loncastuximab tesirine is internalized within the cell, where pyrrolobenzodiazepine is released, promoting cell death.^71^

The phase two, single‐arm LOTIS‐2 trial (NCT03589469) investigated loncastuximab tesirine in patients with R/R DLBCL or high‐grade B‐cell lymphoma (*N* = 145) who had failed at least two prior systemic regimens.^72^, ^73^ After a mean of 4.6 cycles, the ORR was 48.3%, and 25% of patients achieved a CR. Median DOR was 12.6 months for the 70 responders and not reached for patients with CR. ORRs for patients aged ≥75 years, with double‐/triple‐hit DLBCL or with transformed disease were 52.4%, 33.3% and 44.8%, respectively. In 15 patients who received anti‐CD19 chimeric antigen receptor (CAR) T‐cell therapy following treatment with loncastuximab tesirine, the ORR was 46.7%.^73^

The LOTIS‐3 phase 1/2, two‐part, open‐label trial (NCT03684694) is evaluating loncastuximab tesirine in combination with ibrutinib in patients with R/R DLBCL or mantle cell lymphoma.^74^ At data cut‐off, after a median treatment duration of 70 days, the ORR was 62.9% in all patients evaluable for efficacy (*n* = 37), and 58.6% (*n* = 17) in 29 patients with DLBCL. Common grade 3 or higher TEAEs with loncastuximab tesirine include liver enzyme abnormalities and cytopenias.^70^, ^72^

Data from preclinical studies suggest that adding rituximab to an anti‐CD19 pyrrolobenzodiazepine ADC prolongs tumor control, providing a rationale for combining loncastuximab tesirine with rituximab as a treatment for R/R DLBCL.^75^ The phase three, randomized, open‐label, two‐part LOTIS‐5 trial (NCT04384484) is investigating the efficacy of loncastuximab tesirine with rituximab versus rituximab/gemcitabine/oxaliplatin as standard immunochemotherapy in patients with R/R DLBCL.^75^, ^76^

In a pooled safety analysis of loncastuximab tesirine, grade ≥3 neutropenia, thrombocytopenia and anemia occurred in 32.1%, 20.0% and 12.6% of patients with R/R DLBCL, respectively. Most of these events were manageable with dose delays, and did not require dose reduction or discontinuation of treatment.^77^ Liver enzyme elevations are also common with loncastuximab tesirine, which are thought to be due to the pyrrolobenzodiazepine dimer warhead part of the ADC.^78^

Loncastuximab tesirine received FDA approval for use in patients with R/R DLBCL who have received at least two prior systemic therapies.^79^

### 3.4 Anti‐CD19 chimeric antigen receptor T‐cell therapy

Three anti‐CD19 CAR T‐cell therapies are approved for use in R/R DLBCL, after two or more lines of systemic therapy: axicabtagene ciloleucel (axi‐cel) and tisagenlecleucel (tisa‐cel), approved in both Europe and the USA, and lisocabtagene maraleucel (liso‐cel), approved, at present, in the USA only. Although all three therapies use the same single‐chain variable fragment (scFv) derived from clone FMC63, there are differences between them.^80^ For example, axi‐cel contains a CD28 costimulatory domain, while tisa‐cel and liso‐cel each contain a 41‐BB costimulatory domain. However, it is unclear whether these differences affect function, efficacy or safety.^80^ No head‐to‐head comparisons of these therapies have been performed, and clinical trials differ in their designs and patient populations.^81^

In the phase two ZUMA‐1 trial of patients with chemotherapy‐refractory large B‐cell lymphoma treated with a single infusion of axi‐cel (*n* = 101), the ORR was 82% and the CR rate was 54%. After a median of 15.4 months of follow‐up, 42% of patients continued to show a response, and 40% had a CR; at 18 months, the OS was 52%.^82^ At a median of 27.1 months of follow‐up, 83% of patients had an objective response, and 58% had a CR. The median DOR was 11.1 months. Median OS was not reached, and the median PFS was 5.9 months.^83^

The phase two JULIET study of tisa‐cel included patients with R/R DLBCL who had previously received at least two lines of therapy including rituximab and an anthracycline, and were ineligible for ASCT or had disease progression post‐transplant (*N* = 167). In total, 93 patients received one infusion of tisa‐cel, which resulted in a best ORR of 52% (48 patients); 37 (40%) patients had a CR and 11 (12%) had a partial response (PR). Durable responses were observed for up to 18.4 months.^84^

The phase one TRANSCEND NHL 001 study of liso‐cel included heavily pre‐treated patients with R/R large B‐cell lymphomas (*N* = 344). Patients who received at least one dose of liso‐cel (*n* = 269) had received a median of three previous lines of systemic therapy; 67% had chemotherapy‐refractory disease and 44% had never achieved a CR with previous treatment. Median follow‐up was 18.8 months. Of 256 evaluable patients, 186 (73%) achieved an objective response, 136 (53%) had a CR and 51 (20%) had a PR. The response rate at 1 year was 55% in the overall population and 65% in those patients who achieved a CR.^85^

In all three pivotal trials, a significant proportion of patients failed to receive CAR T‐cells: ZUMA‐1, 10/111 (9.0%); JULIET, 54/165 (32.7%); TRANSCEND, 75/344 (21.8%).^82^, ^84^, ^85^

Real‐world studies of anti‐CD19 CAR T‐cell therapies have demonstrated similar favorable outcomes, with comparable rates of durable remission reported to those in the pivotal ZUMA‐1 and JULIET trials, despite the fact that some patients would not have met eligibility criteria for the pivotal trials because of comorbidities, while others presented with more advanced or refractory disease.^86^, ^87^

CAR T‐cells are associated with clinically relevant toxicities (most commonly cytokine‐release syndrome [CRS] and neurotoxicity), which relate primarily to disease burden, the CAR T‐cell dose infused, and patient factors including age and comorbidities. These are typically easily managed and reversible.^81^, ^88^, ^89^ In a retrospective real‐world analysis (*N* = 70), the reported incidence of CRS in patients with R/R DLBCL treated with tisa‐cel or axi‐cel was 85%, with grade ≥3 events occurring in 8% of patients. The incidence of immune cell–associated neurotoxicity syndrome was 28%, including 10% of patients with grade ≥3 events.^86^

Administration of CAR T‐cell therapies must be performed at an authorized center and manufacturing the CAR T‐cells is a lengthy, complex, and costly multistep process.^90^, ^91^ Some manufacturers are attempting to shorten the process by returning fewer cells to patients, favoring in vivo cell expansion. Initially, there was a longer time period from leukapheresis to conditioning chemotherapy in Europe than in the US, due to customs, shipping, and freezing. A higher proportion of patients (up to 97%) then needed bridging therapy, which is associated with poorer outcomes.^86^ The approval of additional CAR T‐cell manufacturing sites by the European Medicines Agency has expanded production capacity in Europe to improve turnaround time.^92^, ^93^ Reported manufacturing failure rates are around 1% for liso‐cel, 1%–3% for axi‐cel, and 7%–9% for tisa‐cel; the latter being notable, given that failure may directly impact on patient outcomes.^84^, ^87^, ^89^, ^94^, ^95^, ^96^

While CAR T‐cell therapies are potentially curative and are therefore a good option for certain patients, they currently have several limitations. In some countries, only a small proportion of patients meet the strict inclusion criteria for treatment. A retrospective analysis conducted at a single center in France found that of 215 patients with R/R DLBCL, primary mediastinal B‐cell lymphomas, or transformed follicular lymphoma for whom a request for CAR‐T was made, 80 (37%) were ultimately deemed eligible for therapy.^97^ Of 74 R/R DLBCL patient cases discussed in Belgium, only 40 patients (54%) were deemed eligible and 38 (51%) received CAR T‐cell therapy (unpublished 2019 data).

Reasons for CAR T‐cell ineligibility include histology, rapid disease progression, need for urgent/bridging therapy, frailty/poor performance status, central nervous system involvement, and reimbursement limitations in individual countries.^97^, ^98^ In some countries, eligibility for CAR T‐cell therapy is based on biological fitness, while in others it is based on chronological age. For example, only patients aged <70 years with an Eastern Cooperative Oncology Group (ECOG) performance status of zero or one would be considered eligible for CAR T‐cell therapy in Italy. Eligibility criteria are less strict in the UK and an increasing number of patients unfit for ASCT are approved for CAR T‐cell therapy. An analysis of data from the UK national CAR T‐cell service found that 250 out of 272 high‐grade lymphoma cases (92%) submitted were approved for treatment, with 163 of the 232 patients (70%) who completed leukapheresis receiving CAR T‐cells.^99^ In patients with highly proliferative disease, particularly where there is bulky disease, there is a need for bridging therapy between leukapheresis and CAR T‐cell therapy to provide disease control.^100^, ^101^, ^102^ A proportion of patients (9%–33% in the pivotal trials) will not be infused because of failure to control the disease, toxicity, or infection‐related complications or nonmeasurable disease before conditioning chemotherapy, despite being deemed eligible to receive CAR T‐cells.^82^, ^84^, ^85^, ^103^ Other factors to consider in decisions regarding CAR T‐cell therapy include patient preference, ability to travel/distance from the treatment center, the presence of a caregiver for recognizing neurotoxicity, disease kinetics, and cost.^87^

Relapse after CAR T‐cell therapy is common, with around half of relapses occurring within the first month in R/R DLBCL.^104^ Risk factors for early progression are two or more extranodal sites, increased C‐reactive protein level, and high total metabolic tumor volume at the time of treatment.^104^ In ZUMA‐1, CD19‐negative relapse occurred in around 30% of patients following axi‐cel therapy.^105^ Though the mechanisms responsible for this are not yet fully clear, it is thought to be due to the emergence of tumor cells with low or no CD19‐antigen expression within the context of targeted removal of antigen‐positive tumor cells.^105^ Local resistance mechanisms within tumors may also play a role in relapse after CAR T‐cell therapy. Lesions at high risk for local failure include high metabolic activity, diameter ≥5 cm, and extranodal disease. It is hypothesized that resistance to CAR T‐cells may occur at the individual lesion level, given that a discordant response to therapy is frequently observed, with some lesions remaining in remission and others progressing.^100^

### 3.5 Other anti‐CD19 therapies in development with potential for use in R/R diffuse large B‐cell lymphoma

Inebilizumab is a humanized, anti‐CD19 monoclonal antibody that targets and depletes CD19‐expressing B cells via ADCC.^106^ In a phase one dose‐escalation study that included six patients with R/R DLBCL, the ORR was 50%; one patient achieved a CR and two patients achieved a PR.^107^

Bicistronic CAR constructs have been engineered using a single vector that encodes two different CARs on the same cell, allowing dual targeting of CD19 and CD20, thereby overcoming the loss of one antigen.^108^

## 4 SEQUENTIAL USE OF ANTI‐CD19 THERAPY

The recent approval of novel, CD19‐directed therapies for DLBCL presents a challenge in determining the optimal sequence and duration of treatments for an individual patient.^1^

Level of CD19 expression is not currently used as a decision tool for anti‐CD19 therapy since CD19 expression is highly conserved, with normal to high levels of expression maintained on nearly 90% of B‐cell lymphomas.^22^ The absence of CD19 expression may be of relevance for treatment decisions following CAR T‐cell therapy, as discussed further below.

With CAR T‐cell therapy, the intent is curative in the setting of refractory disease. With other anti‐CD19 targeted therapies, the goal is to prolong remission and extend survival, with acceptable treatment tolerability. Although CAR T‐cell therapies can achieve durable responses, they are not currently approved for use in the second‐line setting and various barriers prevent their widespread uptake, meaning only selected patients will benefit.^109^

### 4.1 Treatment options for transplant‐ineligible patients with R/R diffuse large B‐cell lymphoma

For transplant‐ineligible patients with R/R DLBCL, no internationally accepted standard has been established. Treatment options for these patients include immunochemotherapy, tafasitamab plus lenalidomide or polatuzumab vedotin with bendamustine and rituximab (where approved).^45^, ^110^

There is currently no overlap between ASCT and CAR T‐cell therapy in the R/R DLBCL treatment algorithm. ASCT is indicated for second‐line use, with CAR T‐cell therapy approved in the third‐line setting. Several trials are currently evaluating CAR T‐cells as second‐line therapy in patients with high‐grade B‐cell lymphoma.^111^, ^112^, ^113^, ^114^ In the ZUMA‐7 trial (NCT03391466), axi‐cel demonstrated a 60% improvement in event‐free survival (EFS; defined as time to disease progression, start of a new therapy, or death from any cause) versus standard of care after approximately 2 years of follow‐up in patients with R/R DLBCL who had failed one initial therapy.^115^, ^116^ Data from an interim analysis of the TRANSFORM trial (NCT03575351) of liso‐cel in patients with high‐risk, second‐line, transplant‐eligible R/R B‐cell NHL demonstrated a significant improvement in EFS versus standard of care.^112^, ^117^ It is anticipated that the use of CAR T‐cell therapy earlier in the treatment sequence may become the new standard for eligible patients in the future.

Some transplant‐ineligible patients may be eligible for CAR T‐cell therapy based on age or biological fitness, with a lower level of fitness required for CAR T‐cell therapy compared with ASCT. These may include patients with a refractory tumor response, intermediate performance status and level of organ function, and a moderate number of comorbidities. In some patients, particularly older adults, evaluation by performance status, cardiac function and glomerular filtration rate may not be sufficient, and a formal assessment of the impact of comorbidities and functional/social decline is recommended to determine potential tolerance to therapy.^118^ Eligibility criteria for these approaches differ between countries. For example, in Spain and the UK, ASCT is typically offered to patients aged <70 years with CAR T‐cell therapy available for patients aged <75 years (Spain) or 70–80 years (UK). In Germany, CAR T‐cell therapy is offered to a similar patient population to that in Spain and the UK, while ASCT is restricted to patients with chemosensitive relapse and those aged <70 years. Furthermore, some patients are ineligible for treatment with CAR‐T cells due to national regulations or lack of access.

Generally, transplant‐ineligible patients who are also ineligible for CAR T‐cells (discussed earlier) could be considered for treatment with tafasitamab plus lenalidomide. These include patients who meet the key inclusion criteria used in the L‐MIND study (relapsed or refractory after one to three systemic regimens, including at least one CD20‐targeting regimen, adequate organ function, ECOG performance status of 0–2, measurable disease).^45^ Although the goal of therapy differs with these two approaches, the 43.9‐month median DOR with tafasitamab plus lenalidomide in L‐MIND is similar to that which can be achieved with CAR T‐cells.^47^ In a long‐term follow‐up of patients with DLBCL treated with anti‐CD19 CAR‐T cells, 48% of treatments resulted in a DOR of over 3 years^119^ However, it is important to note that the characteristics of patients in L‐MIND differed from those in trials of CAR T‐cell therapy.

### 4.2 Does previous use of anti‐CD19 therapy preclude the use of CAR‐T cells or subsequent activity of other anti‐CD19 therapy?

It is not yet known whether the CD19 antigen can be targeted with a different anti‐CD19 therapy after disease progression following a previous CD19‐directed therapy. There are concerns regarding antigen masking and the potential for selection pressure of the prior therapy, which could lead to CD19 antigen escape.^120^ Therefore, it is advisable to assess CD19 expression on a new biopsy. Some pivotal studies of anti‐CD19 CAR T‐cell therapies in aggressive lymphomas excluded patients who had been previously treated with CD19‐targeting therapies. In the real‐world setting, decisions regarding the use of anti‐CD19 therapy prior to CAR T‐cell therapy are made by the physician, taking previous lines of therapy into consideration. In some European countries (e.g., Germany and the UK), CD19 levels are measured prior to CAR T‐cell therapy in patients who have previously received CD19‐targeting therapy.

Evidence from preliminary studies suggests that prior treatment with anti‐CD19 therapies in R/R DLBCL may not preclude the use of anti‐CD19 CAR T‐cells.^120^, ^121^, ^122^ However, these observations are limited by small sample sizes, with heterogeneity in patient populations and use of prior therapies. In a study of 14 patients with DLBCL relapsing or progressing after loncastuximab tesirine treatment (CD19‐positive, *n* = 10; not checked, *n* = 4 after treatment failure), and subsequently undergoing CD19‐directed CAR T‐cell therapy, the best response at 3 months included six patients (43%) with a CR and one patient (7%) with a PR; the ORR was 50%.^121^ In a *post hoc* analysis of 12 patients with R/R aggressive B‐NHL in the TRANSCEND study who had received anti‐CD19 therapy prior to liso‐cel therapy (where subsequent biopsy showed CD19‐positive lymphoma), the ORR was 92% (11 patients); 6 patients (50%) achieved a CR and 5 (42%) achieved a PR; 5 patients had a DOR ≥9 months^122^

A case was reported of a 59‐year‐old female patient in the L‐MIND trial with stable disease following 5 months of treatment with tafasitamab‐lenalidomide, with subsequent progression. After 4 cycles of salvage chemotherapy with rituximab, gemcitabine, and oxaliplatin, the patient underwent CAR T‐cell therapy with axi‐cel with a CR, and achieved sustained remission for nearly 1 year^123^ In an analysis of CD19 expression in tumor lymph node biopsies from six patients with R/R DLBCL treated with tafasitamab plus lenalidomide in the L‐MIND study, immunohistochemistry showed distinct CD19 expression in all 12 pre‐ and post‐treatment biopsies, independent of treatment duration/response, or potential residual tafasitamab exposure. Furthermore, CD19 expression levels following tafasitamab treatment were similar to pre‐treatment baseline levels, suggesting that CD19 expression is maintained after tafasitamab therapy. These results may provide a rationale for the subsequent use of anti‐CD19 CAR T‐cell therapy in this patient population.^124^ These findings warrant further research, given that parental antibody clones of tafasitamab (4G7) and most CAR T‐cell therapies (FMC63) have been found to target overlapping, yet distinct, epitopes of CD19 which are encoded by exon 2 and centered around residue R144 (exon 2 encodes a portion of the extracellular domain of the transmembrane protein).^41^, ^125^, ^126^, ^127^

Phase one data from 14 patients with chronic lymphocytic leukemia showed that CD19 expression levels were maintained on the surface of CD24‐positive B cells following tafasitamab treatment.^128^ Preclinical evidence indicates that exposure to tafasitamab does not impair subsequent anti‐CD19 CAR T‐cell binding in vitro. Various cell lines including DLBCL were incubated with tafasitamab to saturate the CD19 antigens. CD19 saturation did not affect CAR T‐cell effector functions such as cytokine production, degranulation, proliferation, or antigen‐specific killing.^129^

Evidence suggests that low or undetectable CD19 expression levels may be adequate for effective anti‐CD19 CAR T‐cell therapy. In an analysis of CD19 expression in pre‐infusion biopsies taken from patients in the JULIET trial of tisa‐cel, similar response rates were observed irrespective of the level of CD19 expression.^84^

CD19 status prior to CAR T‐cell therapy is not assessed in some countries, while in others CD19‐negative status is an exclusion criterion. Immunohistochemistry is most commonly used for CD19 analysis, but may not be a reliable method of assessment. Different CD19 antibodies have distinct epitopes yet most laboratories use only one CD19 antibody for immunohistochemistry, which could yield false negative results. Furthermore, unlike flow cytometry, CD19 staining by immunohistochemistry is not standardized and is highly variable between laboratories.

### 4.3 Use of anti‐CD19 therapies as bridging therapy

The optimal bridging therapy strategy between leukapheresis and CAR T‐cell therapy remains to be determined. Currently, the goal is to minimize total metabolic tumor volumes with the use of a short bridge during CAR T‐cell manufacturing. However, approaches are likely to change as new data become available.

The use of bridging therapy was an exclusion criterion in the ZUMA‐1 trial of axi‐cel but was allowed in the JULIET trial of tisa‐cel; however, not all patients received it. While evidence is lacking, we currently advise against the use of anti‐CD19 therapy before potentially curative CAR T‐cell therapy, due to the risk of downregulating the antigen.

### 4.4 Use of anti‐CD19 therapy following CAR T‐cell therapy

It is estimated that 30%–60% of patients will progress after CAR T‐cell therapy, with poor outcomes.^130^ Current options for these patients are entry into a clinical trial (e.g., of a bispecific antibody), tafasitamab plus lenalidomide or polatuzumab vedotin plus bendamustine and rituximab (where approved), or palliative oral chemotherapy. If CAR T‐cell therapy is approved in the second‐line setting, it might be estimated that around 35% of patients would be ‘cured’ and 65% would be available for salvage therapy; of this latter group, 30% of patients will lose the CD19 antigen and the remainder will have CD19 expression detectable by immunohistochemistry and could be considered for anti‐CD19 immunotherapy.

## 5 CONCLUSION

The early‐stage expression and efficient internalization of CD19 make it an attractive therapeutic target compared with CD20.^35^ CD19 has demonstrated value as a target in B‐cell NHL, particularly in patients with relapsed or refractory disease, as demonstrated by the efficacy profile of the currently available anti‐CD19 therapies. To date, evidence and knowledge about the sequencing of anti‐CD19 therapies are limited. Collection of prospective data will be essential to provide evidence upon which to base treatment decisions. Further research is also needed into the optimum duration of anti‐CD19 therapy. Selection of the most appropriate treatment strategy should be based on individual patient characteristics (e.g., age, comorbidities, presence of very aggressive lymphoma) and the goal of therapy.

## CONFLICTS OF INTEREST

Sarah Bailly has acted as a speaker for Abbvie and Novartis; and as an advisor for AbbVie, Bristol‐Myers Squibb/Celgene, Janssen, Novartis, Roche, and Takeda.

Guillaume Cartron has acted as a consultant for Bristol‐Myers Squibb/Celgene, MedXcell, Ownards, and Roche; and has received honoraria from AbbVie, Bristol‐Myers Squibb/Celgene, Incyte, Gilead, Jansen, Roche, Novartis, Sanofi, and Takeda.

Sridhar Chaganti has acted as an advisor and consultant and has received meeting attendance support from Atara Bio, Bristol‐Myers Squibb/Celgene, Incyte, Kite Gilead, Novartis, Roche, and Takeda.

Raul Córdoba has acted as a speaker for Abbvie, Astra‐Zeneca, Bristol‐Myers Squibb/Celgene, Janssen, Kite, Roche, and Takeda; and acted as an advisor for Abbvie, ADC Therapeutics, Astra‐Zeneca, Beigene, Bristol‐Myers Squibb/Celgene, Genmab, Incyte, Janssen, Kite, Kyowa‐Kirin, Roche, and Takeda; he has also reserved research grants from Pfizer.

Paolo Corradini has acted as a speaker for Abbvie, Bristol‐Myers Squibb, Kite Gilead, and Novartis; and acted as an advisor for Beigene, Bristol‐Myers Squibb, Kite Gilead, Novartis, Roche, Servier, and Takeda.

Johannes Düll has acted as a speaker and advisor for Incyte and Morphosys.

Isacco Ferrarini has acted as a speaker for Abbvie.

Wendy Osborne has acted as a speaker for Abbvie, Incyte, Kite Gilead, Kyowa Kirin, Novartis, Pfizer, Roche, and Takeda; and acted as an advisor for Autolus Therapeutics, Beigene, Kite Gilead, MSD, Novartis, Roche, Servier, Syneos, and Takeda. She has received travel support from Bristol‐Myers Squibb, Novartis, Pfizer, Roche, and Takeda.

Andreas Rosenwald has no conflicts of interest.

Juan‐Manuel Sancho has acted as a speaker for Bristol‐Myers Squibb/Celgene, Gilead/Kite, Janssen, Novartis, Roche, Servier, and Takeda; and acted as an advisor for Bristol‐Myers Squibb/Celgene, Gilead/Kite, Incyte, Janssen, Novartis, Lilly, Beigene, and Roche.

Hervé Tilly has acted as speaker for Roche and Servier; and acted as an advisor for Astra‐Zeneca, Incyte, Janssen‐Cilag, and Karyopharm.

Eric Van Den Neste has acted as a consultant for Celgene and Novartis.

Andreas Viardot has acted as a speaker for Amgen, Astra‐Zeneca, Bristol‐Myers Squibb/Celgene, Kite, Novartis, and Roche; and as an advisor for Amgen, Bristol‐Myers Squibb/Celgene, Kite, Novartis, and Roche.

Carlo Visco has acted as a consultant or speaker bureau member for AbbVie, Gentili, Gilead, Incyte, Janssen‐Cilag, Pfizer, and Servier; and as an advisor for AbbVie, Gentili, Gilead, Janssen‐Cilag, Novartis, and Roche.

## AUTHOR CONTRIBUTIONS

Sarah Bailly, Guillaume Cartron, Sridhar Chaganti, Raul Córdoba, Paolo Corradini, Johannes Düll, Isacco Ferrarini, Wendy Osborne, Andreas Rosenwald, Juan‐Manuel Sancho, Hervé Tilly, Eric Van Den Neste, Andreas Viardot, and Carlo Visco contributed to the conception and design, analysis and interpretation of data, and drafting and critical revision of the manuscript; provided final approval of the manuscript for submission and publication; and agreed to be accountable for all aspects of the manuscript in ensuring that questions related to the accuracy or integrity of any part of the manuscript are appropriately investigated and resolved.

### PEER REVIEW

The peer review history for this article is available at https://publons.com/publon/10.1002/hon.3013.

## DATA SHARING

Data sharing not applicable to this article as no datasets were generated or analyzed during the current study.

## Data Availability

The author has provided the required Data Availability Statement, and if applicable, included functional and accurate links to said data therein.
